# Development and Validation of a Nomogram for Preoperative Prediction of Localization of Neonatal Gastrointestinal Perforation

**DOI:** 10.3389/fped.2021.754623

**Published:** 2021-11-02

**Authors:** Yao Huang, Yuhua Wu, Dongmei Jin, Qing Tang, Peng Yuan, Qi Lu

**Affiliations:** ^1^Department of Neonatology, Children's Hospital of Chongqing Medical University, National Clinical Research Center for Child Health and Disorders, Ministry of Education Key Laboratory of Child Development and Disorders, Chongqing Key Laboratory of Pediatrics, Chongqing, China; ^2^Department of Neonatology, The General Hospital of Ningxia Medical University, Ningxia, China; ^3^Department of Neonatology, First Affiliated Hospital of Harbin Medical University, Harbin, China; ^4^Department of Neonatal Surgery, Children's Hospital of Chongqing Medical University, Chongqing, China

**Keywords:** neonate, gastrointestinal perforation, nomogram model, perforation site, shock, sepsis

## Abstract

**Background:** Information regarding the localization of gastrointestinal perforation is crucial for the following surgical procedure. This study was to determine the key indicators and develop a prediction model for the localization in neonates with gastrointestinal perforation.

**Methods:** A nomogram to predict the location of neonatal gastrointestinal perforation was developed using a cohort of patients who underwent surgery between July 2009 and May 2021. Baseline variables were analyzed using logistics regression and nomogram developed using significant predictors. The predictive performance of the nomogram was assessed by the concordance index (C-index), calibration curve, and area under the receiver operating characteristic (ROC) curve (AUC). The nomogram was further validated in an integrated external cohort.

**Results:** We investigated the data of 201 patients, of which 65 (32.3%) were confirmed with upper gastrointestinal perforation by surgery. Multivariate logistic regression analysis identified the following as independent predictors: preterm [OR: 5.014 (1.492–18.922)], time of onset [OR: 0.705 (0.582–0.829)], preoperative hemoglobin [OR:1.017 (1.001–1.033)], bloody stool: No [OR: 4.860 (1.270–23.588)], shock [OR: 5.790 (1.683–22.455)] and sepsis: No [OR 3.044 (1.124–8.581)]. Furthermore, the nomogram was effective in predicting the perforation site, with an AUC of 0.876 [95% confidence interval (CI): 0.830–0.923]. Internal validation showed that the average AUC was 0.861. Additionally, the model achieved satisfactory discrimination (AUC, 0.900; 95% CI, 0.826–0.974) and calibration (Hosmer-Lemeshow test, *P* = 0.4802) in external validation.

**Conclusions:** The nomogram based on the six factors revealed good discrimination and calibration, suggesting good clinical utility. The nomogram could help surgeons predict the location of gastrointestinal perforation before surgery to make a surgical plan.

## Introduction

Neonatal gastrointestinal perforation (GIP) has significant morbidity and mortality, which poses a significant challenge for the pediatric surgical team (1–6).

Gastrointestinal perforations commonly result in generalized secondary peritonitis (7, 8). Despite advances in neonatal intensive care, antimicrobial therapy, parenteral nutrition, operative, and anesthetic techniques, neonatal mortality remains high (9). A prompt diagnosis is life-saving, and surgical source control must be placed on top of the therapeutic priority list for all patients with generalized secondary peritonitis (10–12). Byun et al. have found that the time between symptoms and surgical intervention was the only prognostic factor for survival (13). Predicting the location of the perforation before surgery will help shorten the time from the onset to the surgical source control. This leads to a reduction in the total cost of treatment and reduces the operation time caused by preoperative planning, therefore, the time of anesthesia and the risk of infection.

Imaging methods such as abdominal X-ray, ultrasound, and computerized tomography (CT) are commonly used to diagnose GIP. However, most of these methods have some limitations. The detection of free intraperitoneal gas on abdominal X-rays is the most definitive sign of perforation (14). However, abdominal X-rays have limited ability to distinguish the location of perforation. Some studies have proved that ultrasound has a certain value in predicting the location of gastrointestinal perforation. However, the accuracy is closely dependent on the experience of sonographers and the quality of ultrasound equipment. CT has become the preferred method for predicting the location of gastrointestinal perforation (15, 16). However, CT still has limitations, such as CT having a potential risk of radiation exposure and is relatively expensive and time-consuming.

In this study, we aimed to build a diagnostic tool that incorporated the clinical factors and serological biomarkers for early identification of the location of gastrointestinal perforation. This could provide useful information to clinicians for the appropriate management and surgical planning.

## Materials and Methods

### Study Design and Participants

A retrospective review of the clinical records of neonates who underwent surgical treatment for GIP at the Children's Hospital of Chongqing Medical University between July 2009 and May 2021 was performed and used to develop the prediction model. The validation data set comprised neonates with GIP between January 2015 and March 2021 at the First Affiliated Hospital of Harbin Medical University and General Hospital of Ningxia Medical University. All patients received standard of care therapy. This study was approved by the institutional review board of each hospital (No. 2020289). All data were encoded and used anonymously. Verbal informed consent and written informed consent were obtained from all participants' parents/guardians.

### Inclusion and Exclusion Criteria

Patients were first screened for eligibility based on the following criteria: (1) Age at onset ≤ 30 d; (2) Gastrointestinal perforation was found during the operation.

Exclusion criteria: (1) The treatment before entering our hospital is unknown; (2) Neonates with major congenital structural or chromosomal anomalies; (3) Neonates without complete records.

### Data Collection

Data on patient demographics (gestational age, sex, birth weight, pregnancy complications, mode of delivery, multiple pregnancies, number of pregnancies, number of deliveries, Apgar scores at 1 min or 5 min, feeding mode, and time of onset), preoperative laboratory findings (leukocyte count, neutrophil count, hemoglobin, platelet count, C-reactive protein, blood pH, serum lactic acid, and sodium concentration), clinical manifestations (absent bowel sounds, prominent abdominal veins, abdominal distension, abdominal erythema, vomiting, bloody stool, and fever), preoperative complications (shock and sepsis), and the location of gastrointestinal perforation were abstracted from each patient record. For this study, complete-case analysis was undertaken; patients who had data missing for any of the predictive factors in the nomogram were excluded.

### Definitions

GIP was defined as the destruction of integrity of the digestive tract. Patients were divided into upper and lower GIP groups according to the perforation site (above or below Treitz ligament). Preterm- and term-born were defined as birth at <37 and ≥37 weeks' gestation, respectively. Fever was defined as an axillary temperature of at least 37.3°C. Hyponatremia was defined as a serum sodium concentration (Na^+^) < 130 mEq/L (17). Bloody stool was defined as visible blood in the stool. In this study, sepsis and shock were defined according to Pediatric Sepsis Consensus (PSC) criteria (18).

### Statistical Analysis

Categorical variables were presented as frequency rates and percentages, and continuous variables were expressed as mean ± standard deviation (SD) if they were normally distributed or median (interquartile range [IQR]) if they were not. Proportions for categorical variables were compared using the χ^2^ test or Fisher's exact test. Means for continuous variables were compared using independent group *t*-test when the data were normally distributed.

To develop a nomogram with well-calibration and discrimination for prediction for location of GIP before surgery, a model was built in a training set and then validated in another data set. A logistic regression model was used to construct the nomogram. Variables with *P* < 0.05 in the multivariable analysis were included in the nomogram. The nomogram is based on proportionally converting each regression coefficient in multivariate logistic regression into 0-100 points. The scores of each variable can be added to derive the total score, which corresponds to the prediction probability. The predictive performance of the nomogram was measured by concordance index (C-index) and calibrated with 1,000 bootstrap samples to reduce the overfit bias. An AUC > 0.80 was considered to be an acceptable value. The optimal cut-off value determined by the maximum Youden index was calculated by receiver operating characteristic curve analysis. Calibration curves were plotted to assess the calibration of the model, accompanied by the Hosmer-Lemeshow test. The prediction model was validated in an independent external cohort. All analyses were performed using R version 3.6.1. A *p*-value < 0.05 (two-tailed) was considered to be statistically significant.

## Results

In our study, 14 neonates were excluded because there was no clear record of the perforation site. Data were collected on 201 patients from the Southwest center (Chongqing, China) and 69 from the Northwest center (Ningxia, China) and Northeast center (Harbin, China). Finally, a cohort of 69 patients from North centers was used for external validation of the nomogram. Demographic, preoperative laboratory findings, clinical characteristics and complications of the patients in primary cohorts are summarized in Tables 1–3.

**Table 1:** Baseline demographic of patients.

	**UGP (*n* = 65)**	**LGP (*n* = 136)**	***P*-value**
**Maternal factors**, ***n*** **(%)**			
**Pregnancy complications**			
Preeclampsia	13 (20.0)	17 (12.5)	0.236
Gestational diabetes	10 (15.4)	25 (18.4)	0.745
Hypothyroidism	8 (12.3)	9 (6.6)	0.278
Anemia	12 (18.5)	20 (14.7)	0.635
Gestational cholestasis	3 (4.6)	10 (7.4)	0.666
Cesarean	54 (83.1)	97 (71.3)	0.103
Multiple pregnancy	23 (35.4)	45 (33.1)	0.871
Primegravidity	16 (24.6)	50 (36.8)	0.120
Primipara	26 (40.0)	72 (52.9)	0.117
**Fetal factors**			
Preterm birth, *n* (%)	59 (90.8)	88 (64.7)	<0.001
Birth weight (g)^a^	1940 [1650, 2400]	2160 [1715, 2913]	0.024
Low birth weight, *n* (%)	53 (81.5)	84 (61.8)	0.008
Male gender, *n* (%)	43 (66.2)	78 (57.4)	0.299
Apgar <7 at 5 min	3 (4.6)	5 (3.7)	1
Feeding mode, *n* (%)			0.012
No	26 (40.0)	28 (20.6)	
With formula milk	32 (49.2)	83 (61·0)	
Without formula milk	7 (10.8)	25 (18.4)	
Nasal ventilation, *n* (%)	28 (43.1)	37 (27.2)	0.037
Orotracheal intubation, *n* (%)	21 (32·3)	26 (19.1)	0.059
Prenatal steroids, *n* (%)	25 (38.5)	31 (22.8)	0.032
Time of onset, d^a^	3 [2, 4]	6.5 [3, 10]	<0.001

a *Median and interquartile range*.

**Table 2:** Laboratory findings of patients.

	**UGP (*n* = 65)**	**LGP (*n* = 136)**	***P*-value**
**Laboratory findings**			
WBC count, 10^9^cells/L^a^	7.40 [3.55, 11.17]	7.23 [3.88, 14.06]	0.360
<5 or >20, 10^9^cells/L, *n* (%)	37 (56.9)	72 (52.9)	0.705
Neutrophil count, 10^9^cells/L^a^	5.30 [2.11, 7.87]	4·88 [2.34, 9.28]	0.392
Hemoglobin, g/L^b^	153.71 (29.45)	132.90 (32.02)	<0.001
PLT count, 10^9^cells/L^a^	185.00 [138.00, 251.00]	198.00 [127.75, 286.50]	0.424
CRP, mg/L^a^	5.91 [3.21, 20.00]	29.50 [10.75, 54.25]	<0.001
CRP > 8mg/L, *n* (%)	32 (49.2)	105 (77.2)	<0.001
pH^a^	7.26 [7.16, 7.34]	7.34 [7.25, 7.41]	0.002
pH ≤ 7.2, *n* (%)	21 (32.3)	20 (14.7)	0.007
Lactate, mmol/L^a^	1.90 [1.20, 3.30]	1.40 [1.00, 2.32]	0.004
Lactate > 5mmol/L	10 (15.4)	6 (4.4)	0.016
Sodium, mmol/L^a^	135.60 [133.00, 139.00]	136.00 [134.00, 138.00]	0.670
Hyponatremia, *n* (%)	8 (12.3)	10 (7.4)	0.375

*WBC, white blood cell; n, number of patients; CRP, C-reactive protein*.

a *Median and interquartile range*.

b *Mean and standard deviation*.

**Table 3:** Clinical characteristics and complications of patients.

	**UGP (*n* = 65)**	**LGP (*n* = 136)**	***P*-value**
**Signs and symptoms**, ***n*** **(%)**			
Absent bowel sounds	53 (81.5)	121 (89.0)	0.221
Prominent abdominal veins	22 (33.8)	47 (34.6)	1
Abdominal distension	61 (93.8)	130 (95.6)	0.854
Abdominal erythema	11 (16.9)	32 (23.5)	0.376
Vomiting	18 (27.7)	47 (34.6)	0.417
Bloody stool	5 (7.7)	51 (37.5)	<0.001
Fever	8 (12.3)	33 (24.3)	0.075
**Complications**, ***n*** **(%)**			
Shock	15 (23.1)	14 (10.3)	0.028
Sepsis	33 (50.8)	106 (77.9)	<0.001

Upper gastrointestinal perforations were present in 65 of 201 patients (32.3%) and 20 of 69 patients (29.0%) in the train and validation cohorts, respectively. There was no statistically significant difference in upper gastrointestinal perforation rate between the two cohorts (*P* = 0.605).

In multivariable analysis, preterm, time of onset, hemoglobin level, bloody stool, shock, and sepsis were predictors of gastrointestinal perforation sites (Table 4).

**Table 4:** Univariable and multivariate logistics regression analysis.

	**Univariable OR** **(95% CI)**	***P*-value**	**Multivariable OR** **(95% CI)**	***P*-value**
Preterm birth	5.363 (2.309–14.697)	<0.001	5.014 (1.492–18.922)	0.012
Low birth weight	2.734 (1.370–5.799)	0.006		
Feeding mode				
No	Ref			
With formula milk	0.415 (0.211–0.812)	0.010		
Without formula milk	0.302 (0.105–0.785)	0.018		
Nasal ventilation	2.025 (1.089–3.770)	0.026		
Prenatal steroids	2.107 (1.113–4.024)	0.022		
Time of onset	0.686 (0.590–0.780)	<0.001	0.705 (0.582–0.829)	<0.001
Hemoglobin (g/L)	1.022 (1.012–1.033)	<0.001	1.017 (1.001–1.033)	0.040
CRP ≤ 8mg/L	3.493 (1.869–6.611)	<0.001		
pH ≤ 7.2	2.768 (1.369–5.633)	0.005		
Lac > 5mmol/L	3.940 (1.394,12.080)	0.011		
Bloody stool = No	7.200 (2.953–21.634)	<0.001	4.860 (1.270–23.588)	0.031
Shock	2.614 (1.173–5.873)	0.018	5.790 (1.683–22.455)	0.007
Sepsis = No	3.426 (1.827–6.502)	<0.001	3.044 (1.124–8.581)	0.031

*CI, confidence interval; OR, odd ratio; CRP, C-reactive protein; Lac, Lactate*.

**Figure 1:**
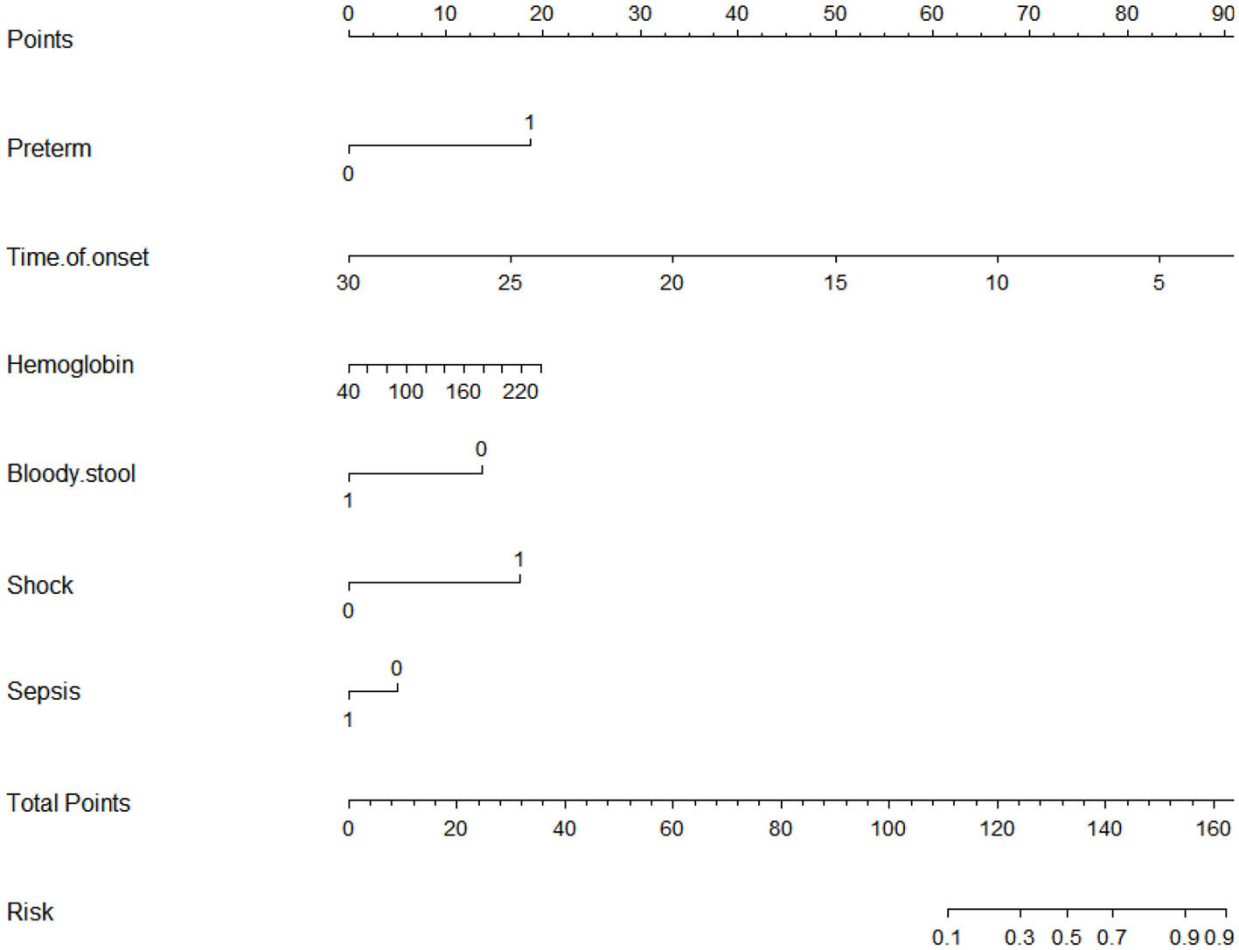
Nomogram for prediction of gastrointestinal sites.

**Figure 2:**
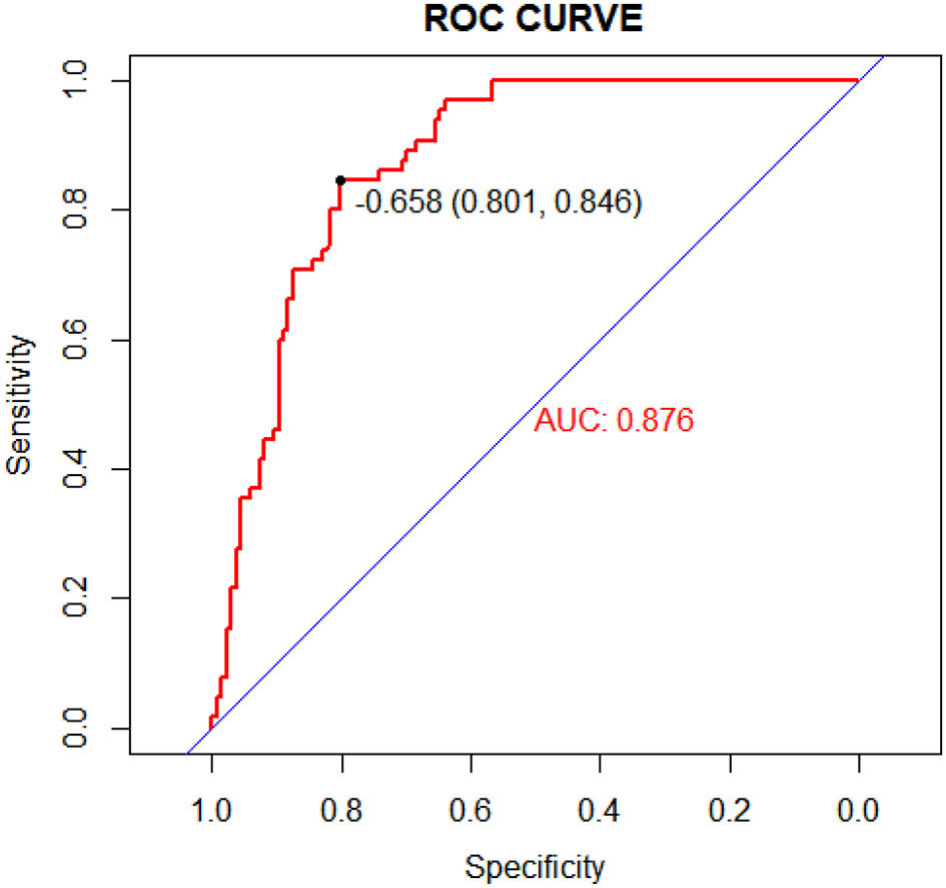
Receiver operating characteristic (ROC) curves of the nomogram model in the training cohort.

**Figure 3:**
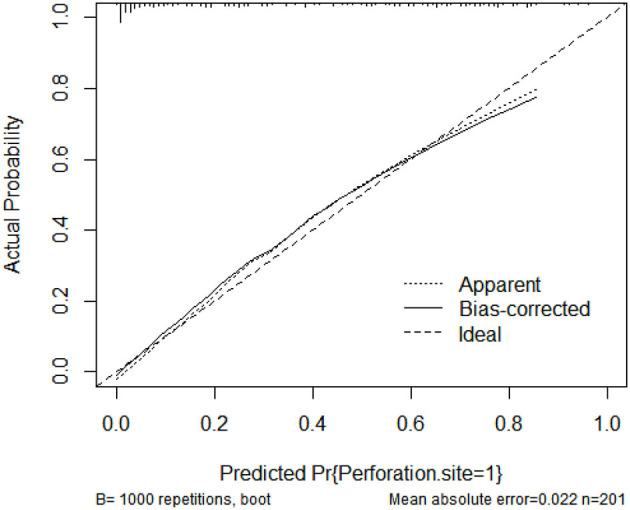
Calibration curves of the nomogram model showing the predicted vs. actual probability for upper GIP (gastrointestinal perforation) in the training cohort.

A nomogram that incorporated these factors was constructed (Figure 1). The C-index for the prediction nomogram was 0.876 (95% CI: 0.804–0·890) for the training cohort and 0.861 *via* bootstrapping validation (Figure 2). The calibration curve for the model showed good agreement between prediction and observation in the training cohort (Figure 3). The Hosmer-Lemeshow test yielded a *P*-value of 0.8977, indicating that the model was well-fitted.

### Development of Nomogram

Logistic regression modeling identified six variables that were associated with upper gastrointestinal perforation: preterm [OR: 5.014 (1.492–18.922); *P* = 0.012], time of onset [OR: 0.705 (0.582–0.829); *P* < 0.001], preoperative hemoglobin [OR: 1.017 (1.001–1.033); *P* = 0.040], bloody stool: No [OR: 4.860 (1.270–23.588); *P* = 0.031], shock [OR: 5.790 (1.683–22.455); *P* = 0.007] and sepsis: No [OR 3.044 (1.124–8.581); *P* = 0.031] (Table 5).

**Table 5:** Predictors for perforation site.

	**OR (95%CI)**	**β coefficient**	***P*-value**
Preterm	5.014 (1.492–18.922)	1.612	0.012
Time of onset	0.705 (0.582–0.829)	−0.348	< 0.001
Hemoglobin	1.017 (1.001–1.033)	0.016	0.040
Bloody stool	4.860 (1.270–23.588)	1.581	0.031
Shock	5.790 (1.683–22.455)	1.756	0.007
Sepsis	3.044 (1.124–8.581)	1.113	0.031

*CI, confidence interval; OR, odds ratio*.

### Validation of Nomogram

**Figure 4:**
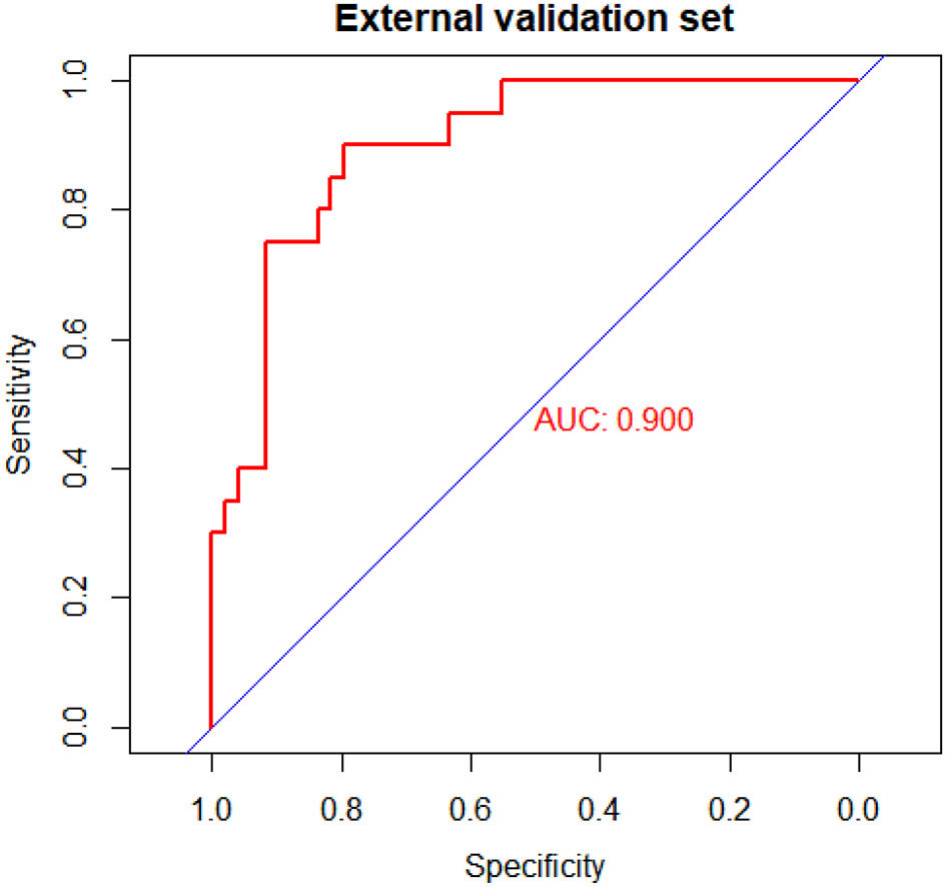
Receiver operating characteristic (ROC) curves of the nomogram model in the validation cohort.

**Figure 5:**
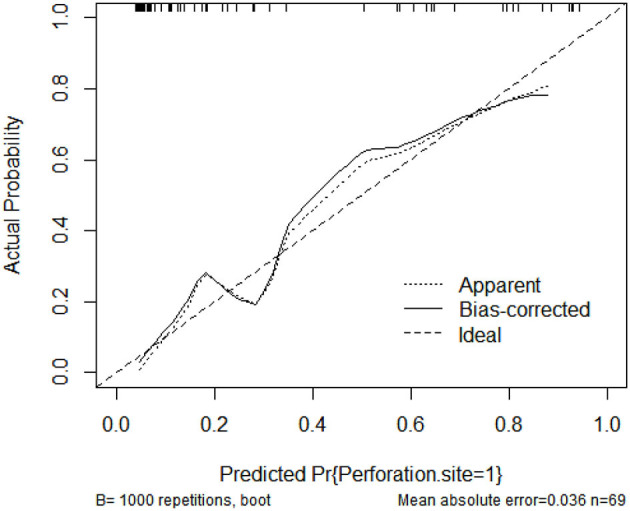
Calibration curves of the nomogram model showing the predicted vs. actual probability for upper GIP (gastrointestinal perforation) in the validation cohort.

The C-index for the prediction nomogram was 0.900 (95% CI: 0.826–0.974) (Figure 4). The calibration curve showed good agreement between prediction and observation for the risk of upper GIP in the validation cohort (Figure 5). The Hosmer-Lemeshow test yielded a non-significant *P*-value of 0.4802.

## Discussion

We developed and validated a prediction nomogram for preoperative prediction of the location of gastrointestinal perforation. The nomogram incorporates six items, including preterm, time of onset, preoperative hemoglobin, bloody stool, sepsis, and shock. The nomogram performed very well to predict the location of GIP in both the training (c-index 0.876) and external validation (c-index 0.900) cohorts. In particular, the calibration plots demonstrated good agreement between the estimated and observed perforation sites. As such, the proposed nomogram may be a helpful clinical tool to clinicians for the appropriate management and surgical planning. This result indicates that this nomogram is expected to be applied to neonates with GIP nationwide.

All predictors included in the nomogram were easy to readily available. It requires no advanced medical equipment and is particularly practical for medical-lacking or developing countries. This approach is less time-consuming, cheaper, and does not require any radiation exposure to the patient.

In the current study, several predictors have been found to be useful in predicting the location of neonatal gastrointestinal perforation.

In our study, the earlier the onset, the greater the possibility of upper GIP. Upper GIP is mostly caused by congenital defects in the musculature of the stomach, so it tends to have an early onset. Saracli et al. have reported that NGP mainly occurs between 2 and 7 days of age (19). Lower GIP is mostly caused by NEC and SIP (20). Preterm perforated-NEC usually occurs between 2 and 8 weeks after birth. And SIP usually presents as an isolated perforation within 10 days of birth (6). Calisti et al. have reported that the mean age of intestinal perforation was 10 days (21).

In our study, we have found that preterm birth was associated with upper GIP. The upper GIP is mostly gastric perforation, and duodenal perforation is extremely rare in newborns (22). In a previous systematic review, 47% of gastric perforation cases were considered idiopathic (23). Neonatal idiopathic gastric perforation is associated with the development of the gastric muscularis and is more likely to occur in premature infants (3). NEC is another major cause of gastric perforation, and it is also related to premature delivery (23).

In our study, a significant decrease in hemoglobin level often indicates intestinal perforation. Lower GIP is often accompanied by primary intestinal lesions, leading to obstacles to the intestinal absorption function. On the other hand, upper GIP is more likely to develop sepsis, causing sepsis-related anemia (24).

The results of this study suggest that the occurrence of preoperative sepsis was associated with lower GIP. Due to the difference in perforation sites and contents, the bacterial load was higher in lower GI than upper GI. When the intestinal integrity is impaired, the microbiota can come in contact with the intestinal mucosa and stimulate inflammation through the immune system (25).

In our study, the occurrence of preoperative shock was associated with upper GIP. Upper GIP is mainly caused by congenital defects in the musculature of the stomach, so the diameter of the perforation is often larger than that of lower GIP. Byun et al. reported that the length of gastric perforation was up to 10 cm in diameter (13). We speculate that this can easily cause a large amount of gastrointestinal fluid to enter the abdominal cavity in a short time, causing chemical peritonitis and even toxic shock.

In our study, we found that bloody stool often indicated lower GIP. Patients with lower GIP often have primary intestinal lesions, which can cause bleeding when the lesions accumulate blood vessels. Lower GI bleeding often manifests as bloody stools, while upper GI bleeding often manifests as melena. This is in line with our observations.

While knowledge of predictors associated with the location of GIP may be helpful, the practical utilization of this information can be challenging in the clinical setting. In turn, predictive model (nomogram) has gained popularity as they are relatively easy to use with a simple graphic that enables the incorporation of multiple relevant clinical predictors that can be applied to individual patients. In addition, in an era of personalized medicine, nomograms directly quantify individual patient risk based on statistically derived predictive variables. The variables used in our predictive nomogram are readily and routinely available (26–28). Importantly, the proposed nomogram to predict the location of GIP performed very well, with a c-index of 0.876 in the training cohort and 0.900 in the validation cohort, as well as excellent calibration. The proposed nomogram may help surgeons predict the location of gastrointestinal perforation before surgery to make a surgical plan.

Our study has some limitations: (1) Although this nomogram was based on Chinese patients alone and may not apply well to Western populations; (2) Although we undertook external validation, this was also performed in a small cohort; (3) The retrospective design and the reliability of the electronic patient records are always associated with limitations; (4) Our hospital is the largest children's medical center in the Southwest China, which may lead to selection bias because we have a higher proportion of critically premature infants; (5) Our model is constructed by comparing data in newborns with UGI and LGI perforations. Therefore, it is not suitable for neonates with no perforations found during surgery. Clearly, our results should be further validated by prospective studies in multicenter clinical trials. Other predictive variables may be included to improve performance of this model.

## Conclusion

Mortality of neonatal gastrointestinal perforation has decreased since development in intensive medical care, but how to predict the location of GIP is still a challenge for surgeons. This study identified predictors for the location of GIP. The nomogram for predicting the location of neonatal gastrointestinal perforation has thus been developed in this study to permit surgeons to make appropriate management and surgical planning.

## Data Availability Statement

The raw data supporting the conclusions of this article will be made available by the authors, without undue reservation. Requests to access these datasets should be directed to Yao Huang, huang199665@qq.com.

## Ethics Statement

This study received approval from the Ethics Committee of Children's Hospital of Chongqing Medical University, First Affiliated Hospital of Harbin Medical University, and the General Hospital of Ningxia Medical University. Written informed consent to participate in this study was provided by the participants' legal guardian/next of kin.

## Author Contributions

YH and QL are the guarantors and take responsibility for the manuscript, including the data and analysis and drafted the manuscript. QL, YH, YW, and DJ conceived and designed the study. YH, YW, DJ, QT, and PY acquired the data. YH and YW analyzed the data. All authors approved the final version for submission.

## Funding

This study was supported by Chongqing Health Committee and Chongqing Science and Technology Bureau [No. 2021ZY023801] and National Special Fund for the Development of Local Science and Technology.

## Conflict of Interest

The authors declare that the research was conducted in the absence of any commercial or financial relationships that could be construed as a potential conflict of interest.

## Publisher's Note

All claims expressed in this article are solely those of the authors and do not necessarily represent those of their affiliated organizations, or those of the publisher, the editors and the reviewers. Any product that may be evaluated in this article, or claim that may be made by its manufacturer, is not guaranteed or endorsed by the publisher.
